# Efficacy of Conbercept in the Treatment of Choroidal Neovascularization Secondary to Pathologic Myopia

**DOI:** 10.3389/fmed.2021.720804

**Published:** 2021-10-21

**Authors:** Hui Lu, Tao Yue, Na Liu, Zuo-Fen Wang, Gai-Xia Zhai, Dong-Ming Mi, Jing Zhang, Shao-Peng Wang

**Affiliations:** ^1^Department of Ophthalmology, Zibo Central Hospital, Zibo, China; ^2^Department of Gerontology, Zibo Central Hospital, Zibo, China; ^3^Jinan No.8 Retired Cadres Rest and Recuperation Home of Shandong Provincial Military Region, Jinan, China

**Keywords:** conbercept, vascular endothelial growth factor, choroidal neovascularization, pathologic myopia, efficacy, VEGF receptor fusion protein

## Abstract

**Purpose:** To observe the clinical efficacy of conbercept in the treatment of choroidal neovascularization (CNV) secondary to pathologic myopia.

**Methods:** We used retrospective analysis of the clinical data of 20 patients (24 eyes) with pathologic myopia choroidal neovascularization (PM-CNV). All patients were treated with intravitreal injection of conbercept 0.5 mg (0.05 ml), a vascular endothelial growth factor (VEGF) receptor fusion protein, and all patients completed at least 6 months of follow-up. Fundus, best corrected visual acuity (BCVA), fundus fluorescein angiography (FFA), optical coherence tomography (OCT), multifocal electroretinogram (mfERG) were assessed before and after treatment. Primary outcome was the functional change in amplitude by mfERG and secondary outcome was the structural change in central macular thickness (CRT) by OCT. The CNV area, leakage of CNV lesions, ocular and systemic adverse events were observed before and after treatment.

**Results:** The BCVA were 64.33 ± 10.83 letters, 65.42 ± 11.24 letters, 67.67 ± 7.07 letters after treatment 1, 3, 6 month, respectively, which showed improvement compared with the baseline (*P* < 0.05). The CRT decreased significantly from 308.50 ± 45.48 μm to 219.63 ± 30.27 μm, 221.33 ± 40.65 μm, 220.96 ± 33.09 μm after treatment 1, 3, 6 month, respectively (*P* < 0.05). The P1 response of mfERG amplitude improved from 40.71 ± 9.69 nv/deg2 to 50.67 ± 9.48 nv/deg2, 54.92 ± 8.45 nv/deg2, 55.67 ± 6.74 nv/deg2 after treatment 1, 3, 6 month, respectively (*P* < 0.05). After 6 months of treatment, the leakage of CNV lesions disappeared in 20 (83.3%) eyes, and the leakage area of CNV lesions was significantly reduced in 4 (16.7%) eyes.

**Conclusion:** The intravitreal injection of conbercept significantly reduced CRT and the CNV area, inhibited the leakage of CNV, improved the BCVA, increased the response of mfERG amplitude, and restored the retinal function. The intravitreal injection of conbercept can change the morphology and function of the macular in PM-CNV, which is safe and effective for the treatment of PM-CNV.

## Background

Pathological myopia (PM) is an important cause of low vision and blindness, especially in Asian populations (1). PM was originally described as high myopia accompanied by characteristic degenerative changes in the sclera, choroid, and retinal pigment epithelium (RPE) with compromised visual function (2). Although there is no universally accepted definition of PM, it is frequently defined as globe elongation and a refractive error of at least 26 diopters (3) and/or axial length of >26.5 mm (4) associated with degenerative changes in the retina (5, 6). The typical pathological features include arc spots, posterior staphyloma, lacquer cracks, Fuchs spots, choroidal atrophy, choroidal neovascularization (CNV), etc., of which the major cause of visual impairment is macular CNV (7). The pathogenesis of PM-CNV is still unclear. The possible mechanisms are choroidal and retinal microcirculation disorders, Bruch membrane rupture, hypoxic and ischemic macular areas, stimulation of the secretion of vascular endothelial growth factor (VEGF) and promotion of CNV breakage through the Bruch membrane into the subretinal space (8). The treatment of PM-CNV includes surgical treatment, drug treatment, laser photocoagulation, etc. (9).

The factors that stimulate pathologic neovascularization are not very clear, but VEGF is considered to be one of the main elements in angiogenesis, and several reports have provided evidence that VEGF-a plays an important role in promoting CNV in PM (10–15). Moreover, studies have shown increased VEGF concentrations in the aqueous humor of patients with CNV secondary to PM compared to controls (16).

Before 2011, the most widely used pharmaceutical agents were monoclonal antibodies that block VEGF-A, which include ranibizumab (Lucentis; Genentech, Inc., South San Francisco, CA, USA) that has been approved by the Food and Drug Administration, and bevacizumab (Avastin; Genentech Inc.) (17–19). Aflibercept (Eylea; Regeneron, Inc., Tarrytown, NJ, USA) was approved as a VEGF receptor fusion protein in 2011 in the USA, and it works as a multitarget VEGF family blocker and binds to isoforms of VEGF-A and VEGF-B as well as placenta growth factor (PlGF) (20). Conbercept (Langmu; Kanghong, Inc., Sichuan, China) is a different VEGF receptor (VEGFR) fusion protein that blocks all isoforms of VEGF-A, VEGF-B, VEGFC, and PlGF, and it has a long half-life in the vitreous body (21). Studies on conbercept have shown that it is an efficient drug for the treatment of neovascular age-related macular degeneration (AMD) (22).

In this study, we observed the clinical efficacy of conbercept for the treatment of PM-CNV to provide guidance for the clinical application of conbercept in PM-CNV.

## Methods

A retrospective analysis of the clinical data from 20 patients (24 eyes) with PM-CNV diagnosed in Department of Ophthalmology, Zibo Central Hospital, from September 2016 to June 2018 was performed. The diagnosis of PM-CNV was confirmed by choroidal neovascular leakage on fluorescein fundus angiography (FFA) and intraretinal or subretinal fluid as determined by optical coherence tomography (OCT). The inclusion criteria were eyes with refractive diopter ≥ 6.0 D or axial length ≥ 26.0 mm, PM-CNV without any treatment, CNV leakage seen with FFA, and a minimum follow-up period of 6 months after the first intravitreal conbercept injections. This study excluded patients with the following criteria: (1) history of ocular trauma; (2) age-related macular degeneration, uveitis, vasculitis, retinal breaks, etc.; (3) history of intraocular injection of drugs and previous photodynamic therapy of PM-CNV; (4) syphilis, tuberculosis and other systemic diseases; (5) refractive interstitial opacity could not be smoothly examined other retinal diseases; (6) patients refused to sign informed consent; and (7) pregnant women. This study was conducted in line with the Helsinki Declaration and was approved by the Ethics Committee of the Zibo Central Hospital, and all patients signed informed consent before surgery.

The intraocular injections were carried out under operating theater conditions. Following topical application of proparacaine, the eyelids, lashes, and conjunctiva were cleaned with 5% povidone iodine. After placement of a speculum to keep the eyelids open, conbercept (0.05 ml/0.5 mg; Chengdu Kang Hong Biotechnology Co., Ltd.) was injected at a distance of 4 mm from the superior temporal quadrant. After the injection, the patient was given a topical antibiotic in the quinolone group to use 4 times each day for a period of 7 days.

The decision to administer further injections was made on an as-needed basis. Each visit incorporated a biomicroscopic examination of the anterior segment, the best-corrected visual acuity (BCVA) was measured using the ETDRS scale, intraocular pressure (IOP) measured using non-contact tonometer (TX-20,Canon, Japen), a fundus examination, and a central retinal thickness (CRT) measurement using OCT (Optovue, Inc., Fremont, CA, USA). The mean central retinal thickness (CRT) was defined as the sum of the thickness of the neurosensory retina and the height of the subretinal fluid. FFA (Spectralis, Heidelberg, Germany) was performed before and 6 months after the intravitreal conbercept injections. The size of CNV was measured in the early-phase FA images using VK-2 5.5.5.0 in the fundus camera (KOWA non-myd 7; Kowa, Japan). Multifocal electroretinogram (mfERG) were examined before and after treatment in 1, 3, 6 month after treatment.

MfERG stimulation was performed with the Visual Evoked Response Imaging System (Retiscan, Wiesbaden/Brandenburg, Germany) equipped with a CRT stimulator. Responses were recorded monocularly using Jet electrode, which was positioned on the inferior cornea along the lid margin and temporally fixed. The pupil of the eye was dilated (≥8 mm) with tropicamide (0.5%, Santen, Japan). Gold-cup reference and surface electrodes were applied on the temple and forehead, respectively. The measurement of mfERG was done as reported (23). Waveform Analysis For each waveform, the amplitude of the first positive peak (P1) of the first-order kernel were determined. P1 amplitude was measured from the trough of the first negative wave to the peak of the positive wave while the implicit time was measured from stimulus onset to the first prominent response peak. MfERG data were grouped into five concentric rings, with ring 1 representing the foveal response.

The following criteria were considered when making a decision about reinjection: persistence or recurrence of subretinal fluid or cystic structures via OCT, an increase in the most recent OCT measurement of CRT of 50 μm or more, incipient CNV, incipient hemorrhage, and a loss of 5 or more letters when compared with the last recorded BCVA.

SPSS version 17.0 was used for statistical analysis. The values are presented as the mean ± SD. The Student's *t*-test or the Mann–Whitney U test was used to determine the significance of the differences in the BCVA, CRT, CNV area and P1 amplitude in ring 1 of mfERG value recorded. A *P*-value < 0.05 considered statistically significant.

## Results

The study included 9 males (11 eyes) and 11 females (13 eyes); the age ranged from 21 to 65 years, with an average value of 40.30 ± 14.27 years; the refractive diopter ranged from −6.5 to −15.5 D, with an average value of −10.58 ± 2.49 D; the average axial length was 28.28 ± 1.53 mm; and there were 12 eyes with the subfoveal type and 12 eyes with the parafoveal type (Table 1).

**Table 1:** Patients bisic characteristics ($\bar{x}$ ± s *n* = 24).

**Characteristics**		**Patients, *N* = 20 (eyes, *N* = 24)**
Gender (eye)	Male	11
	female	13
Age (year)		40.30 ± 14.27
Axis (mm)		28.28 ± 1.53
Course of disease (day)		8.20 ± 5.06
Diopter (D)		–(10.58 ± 2.49)
Type (eye)	Subfoveal	12
	Parafovea	12

**Figure 1:**
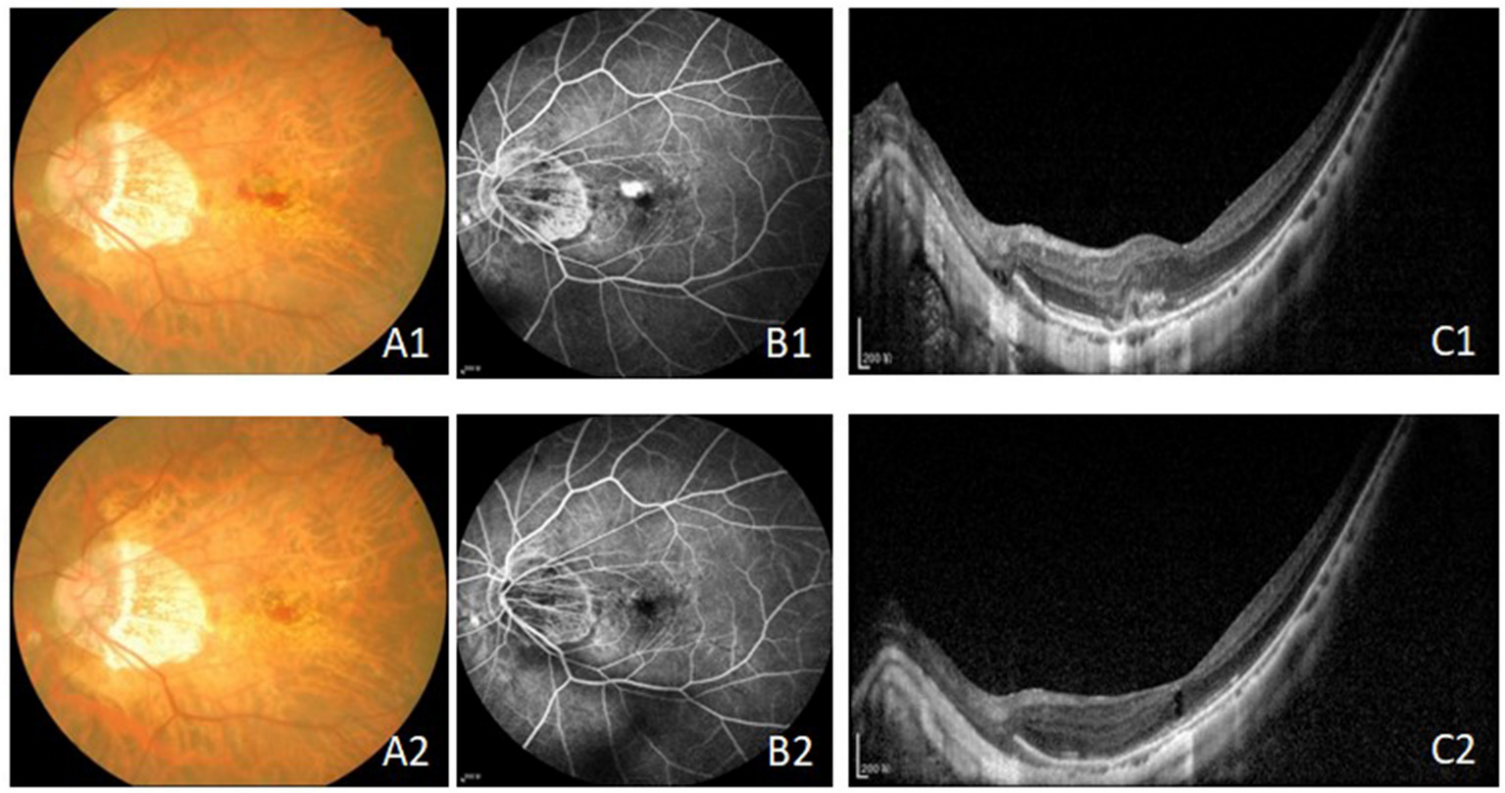
The fundus, FFA and OCT results before **(A1–C1)** and after treatment **(A2–C2)** with intravitreal injection of conbercept in 6 month. The leakage of CNV were inhibited and the CRT and the CNV area were reduced. Bars = 200 μm. FFA, fundus fluorescein angiography; OCT, optical coherence tomography; CRT, central retinal thickness; CNV, choroidal neovascularization.

**Figure 2:**
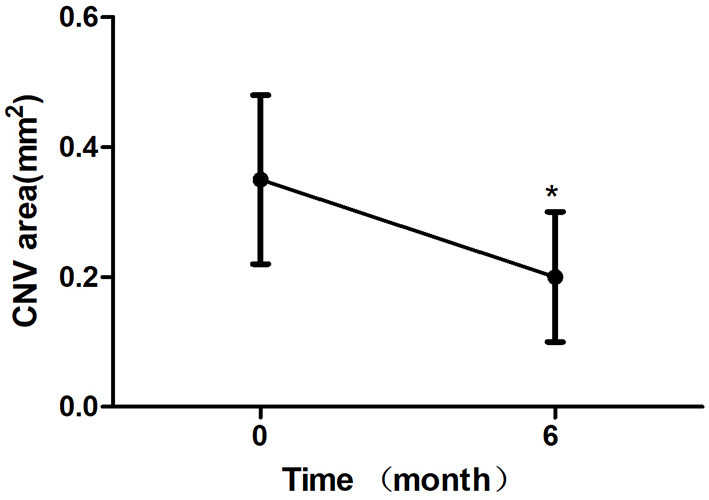
Mean change in choroidal neovascularization (CNV) area over 6 month. The significant reduction of CNV area were observed at 6 month compared with baseline values (*P < 0.05). Error bar represents standard deviation.

All 24 eyes had reduced or stable size of CNV at the last visit (Figure 1). In terms of the mean CNV size, patients with intravitreal conbercept showed a significant reduction of mean CNV size reduction from 0.35 ± 0.13 mm2 at baseline to 0.20 ± 0.10 mm2 at month 6 (*p* < 0.05) (Table 2; Figure 2). An absence of CNV angiographic leakage was observed by FFA in in 20 eyes (83.3%), while slight leakage persisted in 4 eyes (16.7%) at month 6.

**Table 2:** BCVA, CRT, Amp-P1 before and after treatment ($\bar{x}$ ± s *n* = 24).

**Time**	**BCVA (letters)**	**CRT (μm)**	**Amp-P1 (nv/deg2)**	**CNV area (mm2)**
Before treatment	49.96 ± 9.65	308.50 ± 45.48	40.71 ± 9.69	0.35 ± 0.13
1 month	64.33 ± 10.83^*^	219.63 ± 30.27^*^	50.67 ± 9.48^*^	–
3 month	65.42 ± 11.24^*^	221.33 ± 40.65^*^	54.92 ± 8.45^*^	–
6 month	67.67 ± 7.07^*^	220.96 ± 33.09^*^	55.67 ± 6.74^*^	0.20 ± 0.10^*^

* *P < 0.05 compared with the data before treatment*.

**Figure 3:**
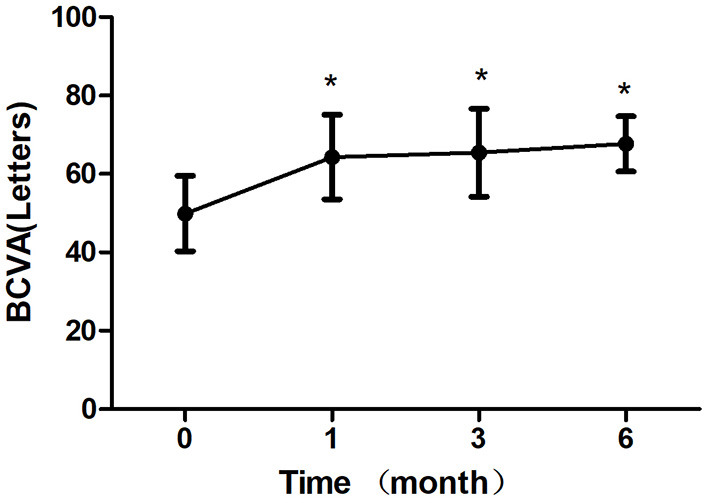
Mean change in best-corrected visual acuity (BCVA) over 6 months. The significant improvements in vision were observed at each followup visit compared with baseline values (*P < 0.05). Error bar represents standard deviation.

**Figure 4:**
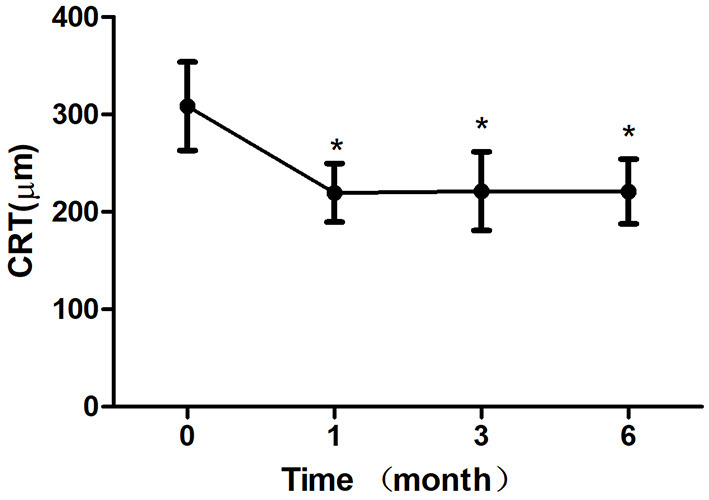
Mean change in central retinal thickness (CRT) over 6 months. The significant improvements in CRT were observed at each follow-up visit compared with baseline values (*P < 0.05). Error bar represents standard deviation.

**Figure 5:**
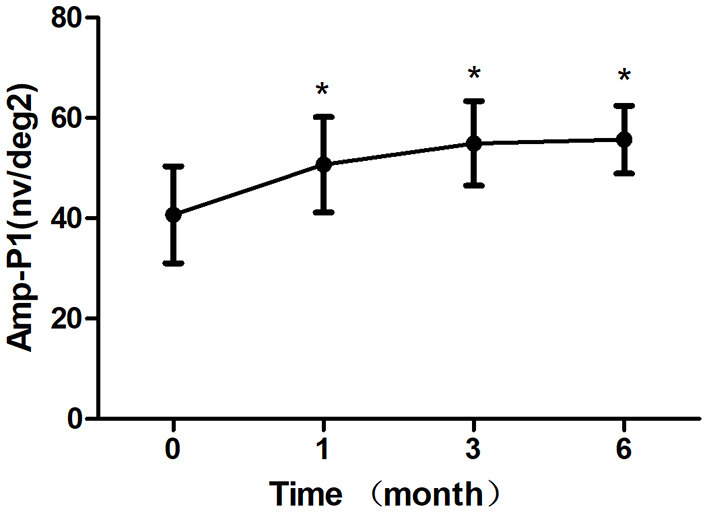
Mean change in amplitude response density in ring 1 of mfERG amplitude (Amp-P1) over 6 months. The significant improvements in Amp-P1 were observed at each follow-up visit compared with baseline values (*P < 0.05). Error bar represents standard deviation.

After 1 month of treatment, 2 eyes (8.33%) demonstrated an increase in the BCVA to <5 letters, 2 eyes (8.33%) increased to 5–10 letters, 16 eyes (66.7%) increased to 10–20 letters, and 4 eyes (16.7%) increased to ≥ 20 letters. The BCVA were 64.33 ± 10.83 letters, 65.42 ± 11.24 letters, 67.67 ± 7.07 letters after treatment 1, 3, 6 month, respectively, which showed improvement compared with the baseline (*P* < 0.05; baseline vs. 1, 3, 6 months) (Table 2; Figure 3). The CRT decreased significantly from 308.50 ± 45.48 μm to 219.63 ± 30.27 μm, 221.33 ± 40.65 μm, 220.96 ± 33.09 μm after treatment 1, 3, 6 month, respectively (*P* < 0.05; baseline vs. 1, 3, 6 months) (Table 2; Figure 4). The P1 response of mfERG amplitude improved from 40.71 ± 9.69 nv/deg2 to 50.67 ± 9.48 nv/deg2, 54.92 ± 8.45 nv/deg2, 55.67 ± 6.74 nv/deg2 after treatment 1, 3, 6 month, respectively (*P* < 0.05; baseline vs. 1, 3, 6 months) (Table 2; Figure 5).

During the follow-up period of 6 months, 17 eyes received 1 intravitreal injection of conbercept, 5 eyes received 2 injections, and 2 eyes received 3 injections.

Subconjunctival hemorrhage occurred in 3 eyes and was absorbed spontaneously 7 days later. The intraocular pressure in 2 eyes increased temporarily and then decreased to normal levels without any treatment. Corneal epithelial injury occurred in 1 eye and was repaired 3 days later. None of the 24 eyes showed obvious ocular or systemic adverse events, such as endophthalmitis, glaucoma, cataract progression, or embolism.

## Discussion

The main treatment for PM-CNV is considered to be photodynamic therapy (8), but the efficacy is poor. Research has reported that (16) the VEGF concentration in the eyes of PM-CNV patients significantly increases, suggesting that the pathogenesis of CNV may be related to VEGF. Conbercept, as a recombinant fusion protein, can inhibit the binding of VEGF to its receptor, thus inhibiting the formation of CNV. Conbercept has many characteristics, such as having many targets, a strong affinity and a long intraocular action time (24). Lu Hang et al. (25) retrospectively observed the clinical data of 62 wet AMD eyes treated with intravitreal injection of conbercept. At the last follow-up, the visual acuity of ETDRS increased to 26.20 letters on average compared with the baseline value. The CRT was significantly thinner than the baseline value, and the difference was statistically significant. In view of the significant efficacy of conbercept in wet AMD patients, this drug may also have a certain effect on PM-CNV.

Some studies (26) have already evaluated the clinical efficacy of conbercept in the treatment of PM-CNV according to the BCVA, CRT and lesion leakage. OCT reflects the regression of macular edema, and FFA reflects the leakage of CNV lesions. How to predict the clinical efficacy of anti-VEGF drugs in the treatment of PM-CNV is difficult. Studies have shown that the anti-VEGF drug response is related to the corrected visual acuity, individual gene type, and size and type of CNV lesions secondary to wet AMD (27).

OCT and FFA can only observe retinal morphology but cannot quantify retinal function. mfERG is currently an effective method for assessing posterior retinal function (28) and has great value for microscopic macular degeneration before morphological changes occur. The P1 response of mfERG amplitude (Amp-P1) values before and after treatment in this study could be sensitive and intuitive for demonstrating that conbercept could improve retinal function of the macular area in PM-CNV. This study innovatively combined the BCVA, CRT and Amp-P1 data to observe the changes in the morphology and function of the macular area in PM-CNV via treatment with conbercept in order to evaluate the efficacy and monitor the changes in the disease (29).

This study was a retrospective analysis of clinical data from 20 patients (24 eyes) with PM-CNV. The results showed that after 1 month ofintravitreal conbercept injection, 2 eyes (8.33%) demonstrated an increase in the BCVA to <5 letters, 2 eyes (8.33%) increased to 5–10 letters, 16 eyes (66.7%) increased to 10–20 letters, and 4 eyes (16.7%) increased to ≥ 20 letters; additionally, there were no significant differences in the BCVA, CRT or Amp-P1 between 1, 3, and 6 months (*P* > 0.05). CNV size and CRT decreased significantly until the end of the follow-up period. This indicates that conbercept is effectively combined with a high concentration of VEGF to improve the BCVA. There were significant differences in the BCVA, CRT and Amp-P1 at each time point after treatment compared with the baseline values (*P* < 0.05). A study showed that (30) the ocular axis of pathologic myopia was >30 mm, and the incidence of fundus lesions was significantly increased. At the same time, we found that 2 eyes with a BCVA increase <5 letters after 1 month of treatment had an axis of >30 mm. In this study, the intraocular pressure increased temporarily in 2 eyes, probably due to the increase in the vitreous cavity content.

A phase III clinical trial of conbercept showed that most patients with retinal disease had a significant increase in visual acuity after 1 month of intravitreal injections and 3 consecutive injections (29, 31). In this study, during the follow-up period of 6 months, 17 eyes received 1 intravitreal injection of conbercept, 5 eyes received 2 injections, and 2 eyes received 3 injections, indicating that the 1+PRN treatment strategies may improve prognosis for most PM-CNV patients and may reduce the economic burden of patients. Advantages of conbercept over other anti-VEGF drugs in PM-CNV need to be observed in the future. A study (32) reported that anti-VEGF drugs increased the risk of cardiovascular and cerebrovascular diseases, but no systemic adverse events were found in this study.

This study found that after conbercept treatment, subretinal fluid and/or intraretinal fluid were significantly absorbed, macular edema was significantly reduced, and corrected visual acuity was significantly improved. We also sensitively and intuitively found that the value of Amp-P1 was significantly improved, so conbercept improved the retinal function of the macular area in PM-CNV. In addition, after 6 months of treatment, the leakage of CNV lesions disappeared in 20 (83.3%) eyes, and the leakage area of CNV lesions was significantly reduced in 4 (16.7%) eyes.

Intravitreal injection of conbercept can reduce the CNV area, macular CRT, inhibit CNV leakage, improve the BCVA, increase the response density of the macular central retina, and restore retinal function in PM-CNV patients. Conbercept can change macular morphology and function in PM-CNV patients, and it is safe and effective for the treatment of PM-CNV. Due to the limited sample size and short observation duration, the long-term efficacy may need long-term observation of large samples.

## Data Availability Statement

The raw data supporting the conclusions of this article will be made available by the authors, without undue reservation.

## Ethics Statement

The studies involving human participants were reviewed and approved by the Ethics Committee of the Zibo Central Hospital. The patients/participants provided their written informed consent to participate in this study.

## Author Contributions

HL and TY wrote the manuscript. NL and D-MM were the patient's operators. G-XZ, Z-FW, and JZ reviewed the literature and contributed to manuscript drafting. S-PW was responsible for the revision of the manuscript for important intellectual content and contributed to diagnosis. All authors issued final approval for the version to be submitted.

## Funding

This work was supported by the Medical and health development plan of Shandong Province, No.2011QW036 and No.2017WS162; and Key Research and Development Plan of Zibo City, Nos. 2019gy010046 and 2019ZC010166.

## Conflict of Interest

The authors declare that the research was conducted in the absence of any commercial or financial relationships that could be construed as a potential conflict of interest.

## Publisher's Note

All claims expressed in this article are solely those of the authors and do not necessarily represent those of their affiliated organizations, or those of the publisher, the editors and the reviewers. Any product that may be evaluated in this article, or claim that may be made by its manufacturer, is not guaranteed or endorsed by the publisher.
